# Effects of temperature, humidity, light, and soil on drug stability in hair: a preliminary study for estimating personal profiles using micro-segmental analysis of corpse hair

**DOI:** 10.1007/s11419-023-00675-9

**Published:** 2023-12-06

**Authors:** Kenji Kuwayama, Hajime Miyaguchi, Tatsuyuki Kanamori, Kenji Tsujikawa, Tadashi Yamamuro, Hiroki Segawa, Yuki Okada, Yuko T. Iwata

**Affiliations:** National Research Institute of Police Science, 6-3-1 Kashiwanoha, Kashiwa, Chiba 277-0882 Japan

**Keywords:** Corpse, Micro-segmental hair analysis, Hay-fever medicine, Personal profile, Environmental condition

## Abstract

**Purpose:**

Micro-segmental hair analysis (MSA), which enables detailed measurement of the distribution of drugs in a single hair strand, is useful for examining the day of death and drug use history of a person. However, corpses are often found in severe environments, such as soil and freezers, which affect the drug contents in hair. Therefore, we examined the effects of temperature, humidity, light, and soil on drug stability in hair as a preliminary study to estimate personal profiles using MSA of corpse hair.

**Methods:**

Four hay-fever medicines (fexofenadine, epinastine, cetirizine, and desloratadine) were used as model drugs to evaluate drug stability in hair. Reference hair strands consistently containing the four medicines along the hair shaft were collected from patients with hay-fever who ingested the medicines daily for 4 months. The hair strands were placed in chambers with controlled temperatures (− 30 to 60 °C) and relative humidities (ca. 18 % and > 90 %), exposed to light (sunlight and artificial lights) or buried in soil (natural soil and compost).

**Results:**

Sunlight and soil greatly decomposed the hair surfaces and decreased the drug contents in hair (up to 37 %). However, all analytes were successfully detected along the hair shaft, reflecting the intake history, even when the hair was exposed to sunlight for 2 weeks and buried in the soil for 2 months.

**Conclusions:**

Although the exposure to sunlight and storage in soil for long times made drug-distribution analysis difficult, MSA could be applied even to hair strands collected from corpses left in severe environments.

**Supplementary Information:**

The online version contains supplementary material available at 10.1007/s11419-023-00675-9.

## Introduction

When corpses of unnatural death are found, an individual is identified, and the cause and time of death are investigated based on the site and anatomical examinations of the corpses. Corpses left in severe environments for long periods can be found in various locations. In murder cases, they are often hidden somewhere, for examples, in freezers, buried in soil, and thrown away in forests and mountains. In accidental cases, corpses can sometimes be found in houses, as in the case of lonely deaths, or in mountains, as in the case of missing persons who got lost. The time of death is generally estimated based on the decomposition state of tissues in the body by anatomical examination. The accuracy of estimation based on anatomical examinations is limited, because the decomposition rates of corpses can vary greatly depending on the environments in which the corpses remain [1–11]. We previously developed a method to estimate the time of death more accurately using micro-segmental analysis (MSA) of corpse hair [12] with an entirely different technique from the anatomical approach [13].

MSA is a method to cut a single hair strand at 0.4-mm interval corresponding to a hair growth length of approximately 1 day [14, 15] and to measure the distribution of drugs in a single hair in detail [16, 17]. In particular, the MSA using internal temporal markers (ITMs) is applied to estimate the day of drug ingestion and death (Supplementary Fig. S1). ITMs are specific compounds which ingested on specific days before hair collection and the positions of ITMs in the analyzed hair strand reflect the drug ingestion day [18–20]. To estimate the day of death, the drug use history during a person’s lifetime, based on information from medical facilities, must be investigated beforehand. When it is recorded that a specific medicine is prescribed on a specific day, it is used as an ITM.

Additionally, the MSA is applicable for estimating personal profiles, because the distribution curves of multiple drugs in a hair strand reflects the drug use history of the person. Even when an individual is not identified based on the site or anatomical examinations of the corpses, the analytical results obtained using MSA can provide valuable information about the person. It is impossible to obtain information of drug use history by the day using conventional segmental hair analysis.

**Fig. 1:**
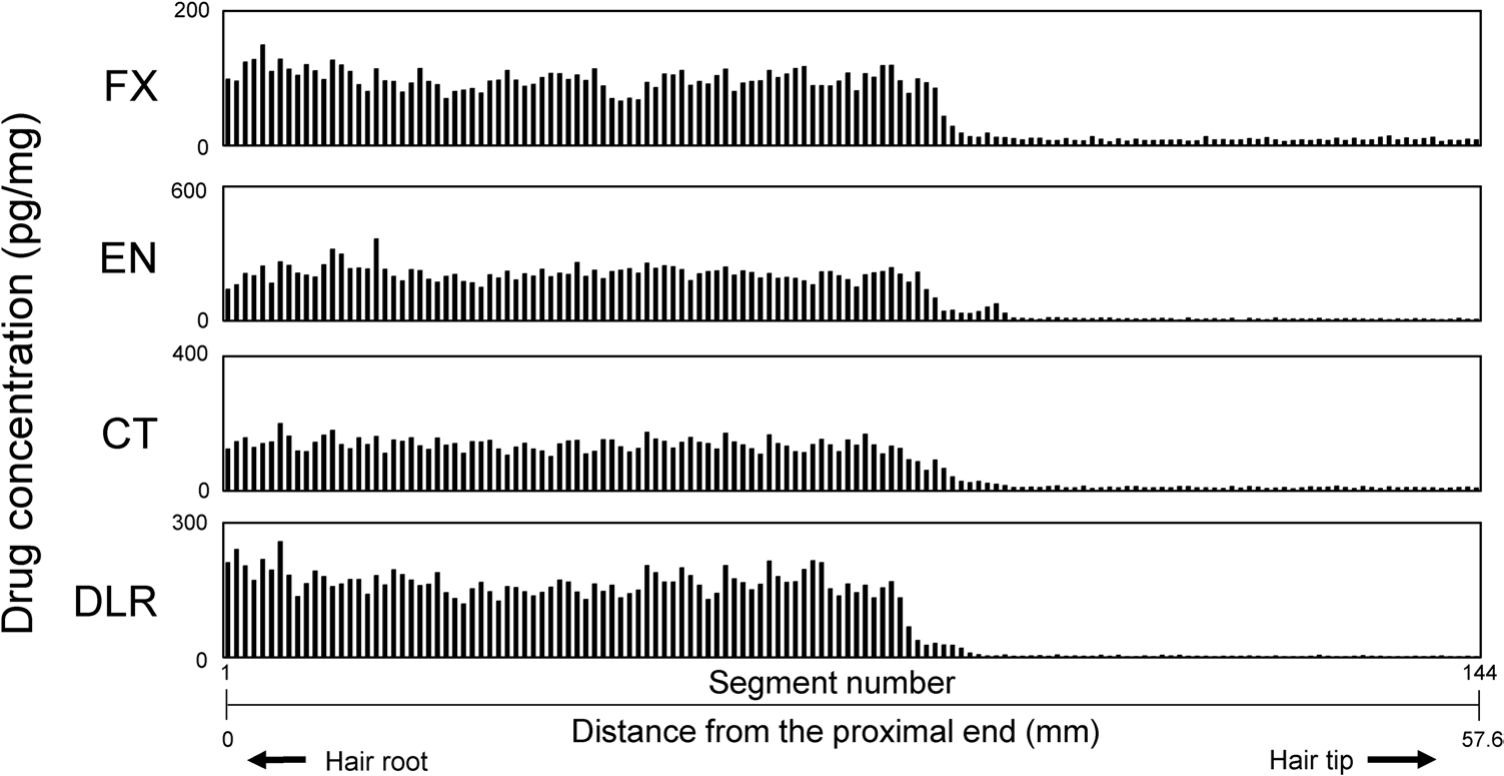
Typical distribution curves in reference hair strand consistently containing the four analytes along the hair shaft. *FX* fexofenadine, *EN* epinastine, *CT* cetirizine, *DLR* desloratadine

It is important to evaluate the applicability of MSA to the hair of corpses left in various situations. We previously examined the drug stability in hair soaked in water, assuming drowned bodies [21]. First, four hay-fever medicines [fexofenadine (FX), epinastine (EN), cetirizine (CT), and desloratadine (DLR)] were selected as model drugs to evaluate drug stability in hair and reference hair strands containing the analytes at constant concentrations along the shaft were prepared (Fig. 1). In the present study, the effects of temperature, humidity, light, and soil on drug stability in hair were evaluated, assuming the corpses remained in severe environments, except water. Moreover, the applicability of MSA to hair strands left in severe environments for a long time was investigated using hair strands collected from a participant who had ingested the four medicines following a determined dosing schedule.

## Materials and methods

### Materials

Four over-the-counter pharmaceutical products for hay-fever, Allegra® FX containing FX, Alesion® 20 containing EN, Stonarhini® Z containing CT, and Claritin® EX containing loratadine were purchased from Hisamitsu Pharmaceutical Co. Inc. (Tosu, Japan), SSP Co., Ltd. (Tokyo, Japan), Sato Pharmaceutical Co., Ltd. (Tokyo, Japan), and Taisho Pharmaceutical Co., Ltd. (Tokyo, Japan), respectively. Chlorpheniramine (CP)-*d*_6_ maleate, which was used as the internal standard, was purchased from Santa Cruz Biotechnology, Inc. (Dallas, TX, USA). The other reagents were purchased from FUJIFILM Wako Pure Chemical Corporation (Osaka, Japan). Acetonitrile, methanol, and ultrapure water were of liquid chromatography/mass spectrometry grade.

### Reference hair strands

Reference hair strands containing drugs consistently along the hair shafts were collected from two participants with hay-fever. They ingested the four hay-fever medicines for self-medication in turn daily for approximately 4 months during the hay-fever season. One participant was an Asian man in his 40 s with straight black hair and the other was an Asian woman in her 30 s with curly black hair. The dosage and ingestion time of the drugs were determined according to the instructions in the medical package inserts (60 mg FX hydrochloride twice a day in the morning and evening, 20 mg EN hydrochloride once a day before bedtime, 10 mg CT hydrochloride once a day before bedtime, and 10 mg loratadine once a day before bedtime). In other words, each hay-fever medicine was ingested at 4-day intervals. A week after the final drug ingestion, tens of black hair strands were cut less than 3 mm above the participant’s scalp from the posterior vertex region using a pair of scissors. The hair strands were stored in the dark at approximately 22 °C until use.

The experiment was performed based on approval by the ethics committee at the National Research Institute of Police Science (No. 2019005, Kashiwa, Japan). Informed consent was obtained from the participants for the experiment.

### Segmentation of reference hair strands

A reference hair strand was segmented into eight regions at 4-mm intervals from the proximal end (Supplementary Fig. S2). The proximal- and distal-end regions were placed in each microtube as no-treatment regions and stored at approximately 22 °C in the dark. Six middle regions were used for temperature, humidity, and light experiments.

### Experiment to evaluate the influence of temperature on drug stability in hair

A 4-mm hair region was placed on a plastic dish and the dish was placed in a freezer (− 30 °C), refrigerator (4 °C), or storage kept at 40 °C or 60 °C for 2 months. Additionally, freezing (− 30 °C) and warming (60 °C) were repeated 10 times at intervals of over 10 min to examine the influence of sudden changes of temperature.

### Experiment to evaluate the influence of humidity on drug stability in hair

A 4-mm hair region was placed on a plastic dish and the dish was placed in a chamber, where the relative humidity was maintained at ca. 18 % using a desiccant or > 90 % using a water bath for predetermined time periods (2 weeks and 2 months).

### Experiment to evaluate the influence of light on drug stability in hair

A 4-mm hair region was placed on a plastic dish and exposed to fluorescent, ultraviolet (UV, 254 nm or 365 nm), and light-emitting diode (LED) lamps and sunlight for predetermined periods (2 weeks and 2 months). In the sunlight experiment, the dish was covered with a plastic lid to prevent rain and wind.

### Experiment to evaluate the influence of soil on drug stability in hair

The reference hair samples were buried in two kinds of soils (“natural soil” and “compost”) outdoors or indoors as shown in Supplementary Fig. S3. “Natural soil” was in bushes, where the soil had never been dug up for over 20 years, within the premises of our institute (Kashiwa, Japan). “Compost” was a soil of a kitchen garden where commercially available compost was laid (Kashiwa, Japan). The distal region, more than 4 cm from the proximal end of a reference hair strand, was placed between the adhesive sheets (JP sheet, Police Science Industry, Ltd. Tokyo, Japan) so that the sample was not lost in the soil. The proximal 1-cm region was cut, placed in a microtube as the no-treatment region, and stored at approximately 22 °C in the dark. A sheet containing the hair sample was buried in soil. In other words, only the middle region (1–4 cm from the proximal end of the reference hair strand) was in contact with soil. For outdoor experiment, a hole approximately 50-cm deep was dug into the bush and kitchen garden. The soil was sieved to remove large materials such as insects and dead leaves. The sieved soil and sheet were placed between two strainers to prevent insects from invading and biting the hair sample during the experiment, placed in a hole, and then filled with soil. Hair samples were left in the soil for determined time periods (2 and 4 months). For the indoor experiment, natural soil and compost were placed in a stainless-steel dish, and a sheet with a hair sample was buried in the soil. The dish was covered with a lid and stored in the dark at approximately 22 °C for 2 months. To prepare sterilized soil, the sieved natural soil was exposed to dry heat at 200 °C for 1 h [22].

### Preparation of hair strands to evaluate influences of severe environmental conditions on drug distributions

Hair strands for drug-distribution measurements were collected from a participant who had ingested the 4 hay-fever medicines for continuous self-medication for 1–10 days, following a determined dosing schedule. Tens of black hair strands were cut from the posterior vertex region near the scalp using a pair of scissors more than half a year after the final drug ingestion. Some hair strands were exposed to sunlight for 2 weeks or 2 months and buried in the kitchen garden (compost) for 2 or 4 months. The remaining hair strands were kept intact in the dark at approximately 22 °C until use. Individual hair strands were subjected to MSA as described in the following section.

#### MSA

Each hair region or a whole hair strand was washed by sonication in an aqueous solution of 1 % sodium dodecyl sulfate for 1 min, followed by alternate washes with water and methanol three times for 1 min each. The hair was attached straight with a double-sided tape on a grid paper which was attached on the stage of a tissue slicer (Stoelting Co., Wood Dale, IL, USA) with a 50 mm micrometer scale (Mitutoyo Corp., Kawasaki, Japan), so that the hair growth direction was aligned at a right angle to the blade [16, 23]. The hair strand was cut at 0.4 mm from the proximal end, and the segment was placed in a 0.1 mL microtube using a tapered cotton swab. The stage was then moved 0.4 mm to the proximal side using the micrometer scale. The procedure consisting of the segmentation, collection, and stage movement was repeated. A mixture of an aqueous solution of 5 mM ammonium acetate containing 0.05 % formic acid (mobile phase A) and acetonitrile (3:1, by vol.) was used as the extraction solution. The extraction solution (100 μL) containing CP-*d*_6_ (4 pg/mL) was added to the tube containing the segment. The sample tube was sonicated at 23 kHz for 10 min and then maintained at 40 °C for 24 h. The supernatant (35 μL) was diluted with mobile phase A (35 μL). The solution (50 μL) was then injected into a liquid chromatograph-tandem mass spectrometer (Waters ACQUITY UPLC I-Class and Xevo TQ-S, Milford, MA, USA). The analytical conditions are summarized in Supplementary Table S1.

### Data analysis

The analytical method was previously validated using spiked hair segments [24], according to the method validation guideline of the Scientific Working Group for Forensic Toxicology [25]. Because loratadine was rapidly metabolized in the body, its metabolite, DLR was targeted in this study. The validation data are summarized in Supplementary Table S2, and the typical chromatograms of the 4 analytes are shown in Supplementary Fig. S4. The coefficients of variation of drug concentration in 0.4-mm segments obtained from the proximal 3.2-cm region in an untreated reference hair strand were less than 20 % in 3 hair strands of each participant. Additionally, it was confirmed for every hair strand that no significant differences in drug concentrations were observed between the proximal and distal-end regions (Fig. S2). Nine 0.4-mm hair segments were prepared from a 4-mm hair region. The drug concentrations in 18 segments from the no-treatment regions (both end regions) in a reference hair strand were averaged. The residual rate of drugs in hair was calculated by dividing the drug concentrations in a 0.4-mm segment from the treated hair regions by the average concentration from the no-treatment regions. In other words, nine residual rates of drug concentrations in the 0.4-mm segments came from a treated hair region. The average residual rate (*n* = 18) was calculated using two strands of hair collected from different participants. There were no large differences in the residual rate between hair strands from two participants in any experimental conditions. A significant difference in the residual rate between the treated and untreated regions was evaluated using Student’s *t*-test. Distribution curves of each analyte in a hair strand were constructed by assessing the drug concentration in each 0.4-mm hair segment, numbered from the proximal to distal ends of the hair strand.

### Environmental parameter measurements and hair surface observation

The temperature, humidity, illuminance, and UV intensity around the samples in the air were monitored using a data logger (TR-74Ui, T&D Corp., Matsumoto, Japan). The temperatures around the sample buried in the soil were monitored using a data logger (Hygrochron, KN Laboratories, Inc., Ibaraki, Japan). The chemical states (pH, nitrate nitrogen, water-soluble phosphate, and water-soluble potassium) of the soil were determined using a soil diagnostic kit (Midori-kun, Fujihara Industry Co., Ltd., Tokyo, Japan). The number of bacteria in the soil was measured using a bacterial testing kit (Biochecker FC, San-ai Obbli Co., Ltd., Tokyo, Japan). The surfaces of hair strands treated under various conditions were observed using a digital microscope (NSH500, Shodensya, Osaka, Japan).

## Results

### Influences of temperature and humidity on drug stability in hair

The drug content in hair hardly changed between  − 30 °C and 40 °C (Table 1). Repetitive sudden changes of temperature from − 30 °C to 60 °C did not also affect the drug contents. DLR decreased up to 54 % in hair strands warmed at 60 °C for 2 months. Excessively low (ca. 18 %) humidity did not indicate drastic decreases of the drug contents in hair. DLR decreased up to 60 % at  > 90 % relative humidity for 2 weeks.

**Table 1:** Effects of temperature, humidity, light, and soil on the drug contents in hair

Condition	Residual rate (%)^*1^			
	FX	EN	CT	DLR
Temperature experiment				
− 30 °C for 2 months	98 ± 12	87 ± 15	88 ± 22	100 ± 15
4 °C for 2 months	112 ± 15	89 ± 15	101 ± 24	119 ± 15
40 °C for 2 months	92 ± 11	94 ± 14	111 ± 27	80 ± 28
60 °C for 2 months	92 ± 12	72 ± 11	109 ± 22	54 ± 14
Cold (− 30 °C) and hot (60 °C)^*2^	92 ± 15	81 ± 14	87 ± 12	84 ± 15
Humidity experiment				
Relative humidity ca. 18 % for 2 weeks	98 ± 13	85 ± 9	92 ± 15	80 ± 20
Relative humidity ca. 18 % for 2 months	88 ± 10	77 ± 11	81 ± 14	76 ± 16
Relative humidity > 90 % for 2 weeks	97 ± 17	80 ± 14	90 ± 19	60 ± 23
Relative humidity > 90 % for 2 months	95 ± 10	84 ± 7	81 ± 15	62 ± 13
Light experiment				
Fluorescent light, 500 lx for 2 weeks	107 ± 15	108 ± 24	91 ± 19	96 ± 20
High-brightness LED, 100 klx for 2 weeks	68 ± 12	122 ± 18	104 ± 16	71 ± 7
254 nm UV lamp, 0.6 mW/cm^2^ for 2 weeks	87 ± 14	119 ± 19	94 ± 20	68 ± 13
365 nm UV lamp, 0.7 mW/cm^2^ for 2 weeks	71 ± 8	104 ± 12	98 ± 19	79 ± 8
Sunlight for 2 weeks^*3^	80 ± 11	71 ± 12	91 ± 24	54 ± 7
Sunlight for 2 months^*3^	42 ± 4	60 ± 10	69 ± 13	37 ± 5
Soil experiment				
Sterilized natural soil, indoors for 2 months	112 ± 11	118 ± 27	98 ± 5	95 ± 18
Natural soil, indoors for 2 months	93 ± 18	119 ± 31	112 ± 20	133 ± 22
Compost, indoors for 2 months	86 ± 14	83 ± 17	104 ± 15	113 ± 32
Natural soil, outdoors for 2 months	102 ± 16	93 ± 18	125 ± 27	69 ± 9
Compost, outdoors for 2 months	93 ± 7	92 ± 8	96 ± 12	79 ± 7
Compost, outdoors for 4 months	68 ± 17	69 ± 20	53 ± 17	67 ± 11

*FX* fexofenadine, *EN* epinastine, *CT* cetirizine, *DLR* desloratadine

^*1^ Residual rate represents the ratio of the drug in treated hair segments to that in hair segments stored in the dark at ca. 22 °C and relative humidity of ca. 30–70 % (no treatment). Values represent the average ± standard deviation of 0.4-mm segments (*n* = 96 for the soil experiment; *n* = 18 for the other experiments) from two individual strands of hair. Underlined values indicate the significant difference compared to the untreated hair strands at the level of *p* < 0.05 using the Student’s *t*-test

^*2^ Hair strands were stored at − 30 °C and 60 °C in intervals of over 10 min 10 times

^*3^ Averages and ranges of temperature, relative humidity, illuminance, and UV intensity during the experiment are described in text

### Influence of light on drug stability in hair

**Fig. 2:**
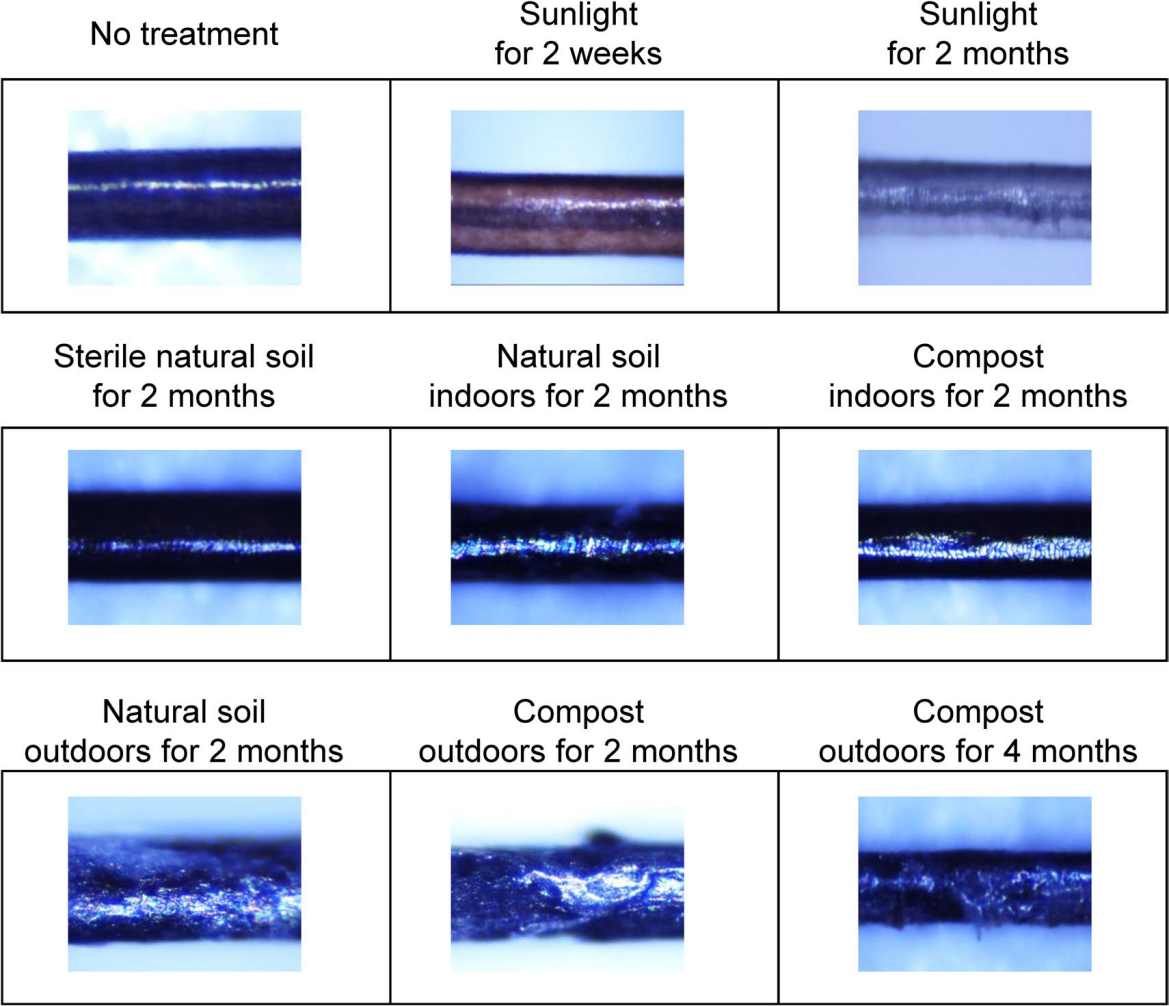
Images of hair strands exposed to sunlight and buried in soil

The general illuminance of fluorescent light in the rooms (approximately 500 lx) did not affect the drug content in the hair (Table 1). The illuminance of the high-brightness LED, which was adjusted to a value close to the maximum illuminance in the sunlight experiment (ca. 100 klx), decreased FX up to 68 %. Both UV lights with short and long wavelengths also decreased DLR up to 68 %. Sunlight significantly decreased the content of all tested drugs in a time-dependent manner. In the sunlight experiment, the temperature, humidity, illuminance, and UV intensity had been monitored for the experimental time periods (2 months). The averages and ranges of outside temperature, relative humidity, illuminance, and UV intensity were 22 °C (− 1.8 to 74 °C), 58 % (1–100 %), 19 klx (0.02–115 klx), and 0.36 mW/cm^2^ (0–2.1 mW/cm^2^), respectively. There were sudden changes in the environmental parameters between the daytime and nighttime (Supplementary Fig. S5). The temperature, illuminance, and UV intensity were the highest a little after noon, while the humidity was comparatively high from dawn to early morning, although the peak height and time of humidity depended on the weather. When the black hair strands were exposed to sunlight, the hair turned brown after 2 weeks and almost white after 2 months (Fig. 2).

### Influence of soil on drug stability in hair

There are two main types of places where corpses are buried in murder cases. One is deserted land around forests and mountains, and the other is a garden or field owned by the suspect. Two types of soil (natural soil and compost) were used in this study. The biological and chemical states of soil samples were similar. The number of bacteria (> 10^5^ colony forming units (CFU)/mL), pH (4.5), and concentrations of nitrate nitrogen (0 mg/L) and water-soluble phosphate (12 mg/L) in the natural soil, measured using assay kits, were the same as those in the compost. The concentration of water-soluble potassium differed between the natural soil (< 3 mg/L) and the compost (7 mg/L). The number of bacteria changed to approximately 10^4^ CFU/mL after both soils were placed indoors in the dark for 2 months. Bacteria were not detected immediately after the natural soil was sterilized with heat, and the number of bacteria increased slightly (< 10 CFU/mL) after the soil was placed indoors in the dark for 2 months. The soil temperatures around the samples in the outdoor experiment were 15 °C on average (the range of 6.5–30 °C) in natural soil and 26 °C on average (the range of 15–55 °C) in compost for the experimental periods (2 months). The surface structures of hair strands buried in the soils differed depending on the environment (Fig. 2). In the no-treatment and sterilized soil, the surfaces were glossy, and light from the coaxial vertical illuminating device of the microscope was reflected straight along the hair shaft. In soils with many bacteria, the reflected light was scattered. Surface structures were destroyed in some places, particularly hair strands left in the soils outdoors. Regardless of the apparent decomposition of the hair surface, the drugs were comparatively retained, even in hair strands buried in the soils for 2 months (Table 1). However, longer storage in soil (4 months) greatly decreased the content of all analytes in the hair.

### Distribution of drugs in hair strands left in severe environments

**Fig. 3:**
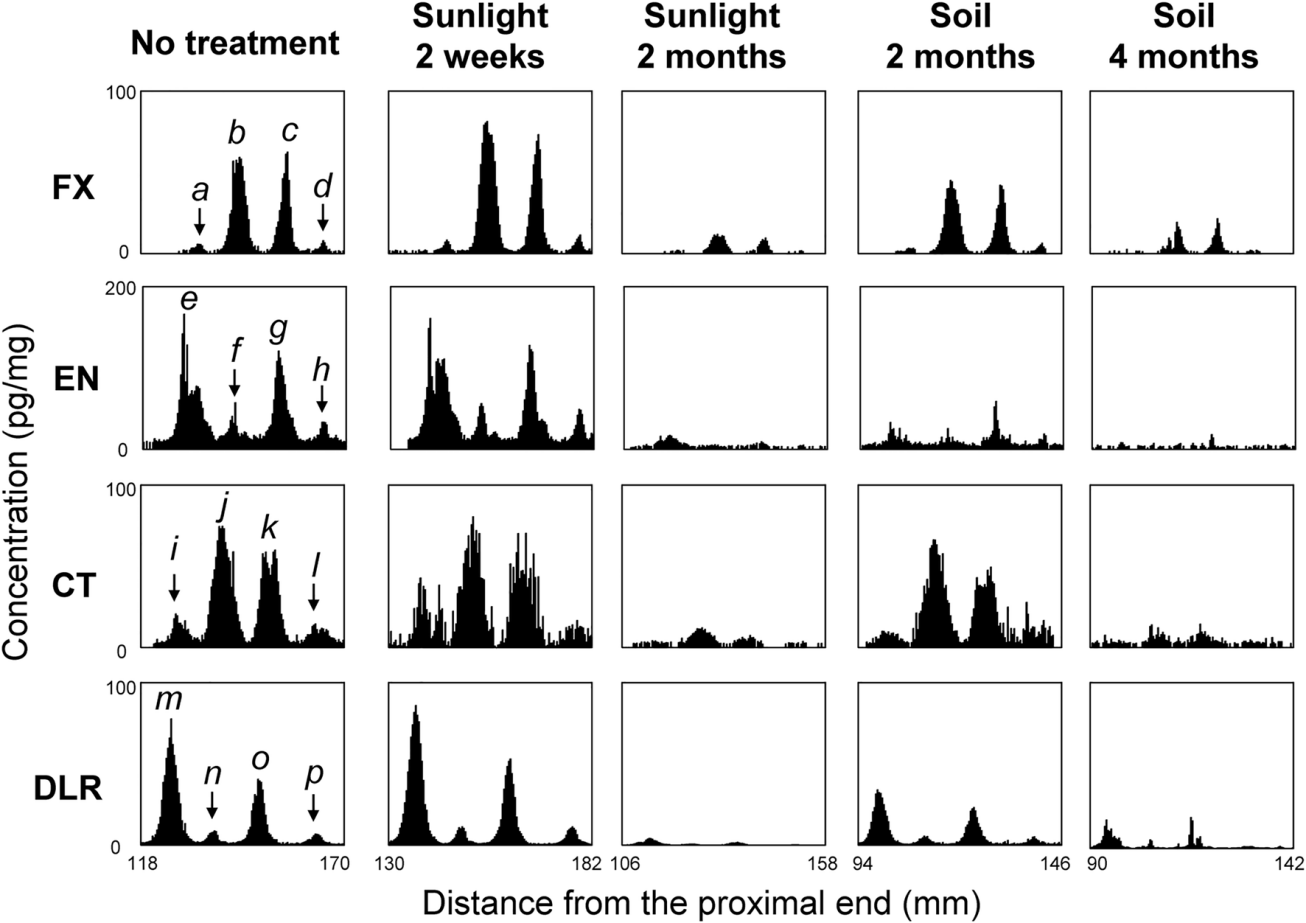
Typical distributions of hay-fever medicines in hair strands exposed to sunlight and buried in soil. One participant ingested four hay-fever medicines continuously (1–10 days) during the hay-fever season. Hair strands were collected approximately 9 months after the final ingestion. Number of days of continuous ingestions corresponding to the peaks are as follows: peaks *a* (1 day), *b* (10 days), *c* (6 days), *d* (1 day), *e* (9 days), *f* (1 day), *g* (5 days), *h* (1 day), *i* (1 day), *j* (9 days), *k* (5 days), *l* (1 day), *m* (9 days), *n* (1 day), *o* (5 days), and *p* (1 day). The drug distributions in strands of hair no. 3, 8, 10, 12, and 14 (see Table 2) are shown from left to right

Hair strands (approximately 20 cm long) collected from a participant who had ingested the four hay-fever medicines following a determined dosing schedule (four cycles of a continuous ingestion for 1–10 days for each medicine) were subjected to MSA (Fig. 3). The drug peaks were detected at specific regions in the untreated hair strand, reflecting the ingestion time (approximately 9 months before hair collection) and the number of days of continuous ingestion (1–10 days). When a whole hair strand was exposed to sunlight for 2 weeks, the distribution curves were similar to those of the untreated hair strand. However, in the hair strand exposed to sunlight for 2 months, the distribution curves had smaller peaks than those in the no-treatment and 2-week sunlight exposure. In contrast, in the hair strand buried in the soil outdoors for 2 months, the distribution curves were similar to those of the untreated hair strand. However, the 4-month of storage in the soil significantly decreased the drug peaks in the distribution curves.

Similarly, several hair strands were subjected to MSA to confirm the reproducibility of drug-distribution curve (Table 2), because the amount of drug uptake differed depending on the activity of individual hair strands [14, 17]. The results showed similar tendencies between hair strands under the same conditions (*n* = 2–5), although large variations were observed between individual hair strands.

**Table 2:** Intensities of drug peaks on the distribution curves in hair strands exposed to sunlight and buried in soil

Hair no	Condition	Maximum concentration (pg/mg)^*1^			
		FX	EN	CT	DLR
1	No treatment	65	177	85	92
2		61	186	80	80
3		58	250	75	143
4		45	240	66	80
5		71	507	119	128
Ave		60 ± 10	272 ± 135	85 ± 21	105 ± 29
6	Under sunlight for 2 weeks	62	697	108	135
7		54	255	69	139
8		75	241	80	159
Ave		64 ± 10	398 ± 259	86 ± 20	144 ± 13
9	Under sunlight for 2 months	2.0	28	5.7	16
10		11	26	12	7.6
Ave		6.4 ± 6.2	27 ± 2	8.9 ± 4.5	12 ± 6
11	In soil for 2 months	33	115	98	59
12		41	88	66	63
13		98	176	103	152
Ave		58 ± 36	126 ± 45	89 ± 20	91 ± 53
14	In soil for 4 months	20	27	14	34
15		17	24	19	42
16		1.2	13	4.5	0.9
Ave		13 ± 10	21 ± 8	12 ± 7	26 ± 22

^*^1 Drug concentrations corresponding to the maximal peak on each distribution curve (for example, peaks *b*, *e*, *j*, and *m* in Fig. 3) were measured. Average values for each condition were calculated. Underlined values indicate the significant difference compared to the untreated hair strands at the level of *p* < 0.05 using the Student’s *t*-test

## Discussion

### Necessity of MSA using a single reference hair strand to evaluate drug stability in hair

MSA is not only indispensable to estimate the day of drug intake but also applicable to estimate the day of death and personal profiles based on the distribution curves of multiple drugs in a corpse hair [23]. In this study, the influences of natural environments on drug stability in hair were evaluated, assuming estimation of personal profiles using hair strands of a corpse left in various situations. As the evaluation method, it is inappropriate to compare the drug concentrations among individual hair strands treated under different conditions, because there are differences in the amount of drug uptake between individual hair strands [14]. Although bulk analysis using multiple hair strands can reduce deviations in the drug concentration, hundreds of hair strands are required to average the drug concentrations in the reference hair samples. In contrast, when a single reference hair strand contains a target drug with a constant concentration along the shaft, segmental analysis of a single hair strand can be applied. In other words, some homogenous hair regions segmented from a single hair strand can be treated under different conditions and compared. If the aim of this study is only to evaluate drug stability in hair, conventional segmental analysis of a single hair strand can be used. The use of MSA is inevitable for estimating personal profiles based on distribution curves of multiple drugs in forensic cases. Therefore, the experimental procedure as shown in Fig. S2 was adopted.

Four hay-fever medicines were used as model compounds for this study. Such antihistamines are not generally important target compounds in forensic cases. However, any medicines can be used as ITMs to estimate the day of hypnotic ingestion in drug-facilitated crimes [19, 20] and become target compounds to estimate personal profiles such as whether the person has hay-fever. Additionally, patients with hay-fever usually ingest antihistamines daily during hay-fever season. Therefore, the four hay-fever medicines were convenient as model drugs to prepare the reference hair strand containing specific drugs consistently along the shaft.

### Influence of temperature on drug stability in hair

We previously demonstrated that DLR decreased in hairs heated at 150 °C or higher for 1 day [26]. DLR tended to be comparatively heat-sensitive for the analytes. However, the four analytes were not significantly decreased even by repetitive sudden changes of temperature from  − 30 °C to 60 °C. The experimental conditions at  − 30 °C and 4 °C were set assuming that corpses were hidden in freezers and refrigerators, or left in cold places such as snowy mountains. Whereas, the experimental conditions at 40 °C and 60 °C were set assuming that corpses were left in temperature-uncontrolled rooms or exposed to sunlight in hot summer. In daily life, we occasionally experience high and low temperatures in tropical and cold areas, respectively.

Temperature management is also important for storing specimens after hair collection. The Society of Hair Testing (SoHT) guidelines state that “hair samples should not be stored in the refrigerator or freezer, since swelling may occur and drug may be lost” [27]. Freezing leads to the formation of ice crystals due to the natural water content of hair. These crystals damage the hair structure leading to a drug loss when thawing. Additionally, sharp temperature changes from cold to warm places cause water vapor in the air to adsorb on hair surfaces, and water penetrating the hair may wash out drugs. Therefore, hair samples should be stored at room temperature just in case, although the contents of the hay-fever medicines used in this study in hair were hardly affected, even by sudden temperature changes.

### Influence of humidity on drug stability in hair

Water vapor in the air tends to adsorb easily onto hair surfaces in highly humid environments. Because corpses left in humid conditions, such as the rainy season, are likely to be damp, some drugs in hair may be washed out by water penetrating the hair. We previously demonstrated that the drugs decreased in hair soaked in water without divalent positive ions such as Ca^2+^ and Mg^2+^ [21]. The effects of humidity on EN and DLR were larger than those on FX and CT, as in the previous study on hair strands soaked in water without divalent positive ions. EN and DLR are basic compounds, while FX and CT are zwitterions containing both amino and carboxylic moieties. Water molecules in hair would contribute to the binding states between drugs (especially basic compounds) and hair tissue. The SoHT guidelines state that “hair samples that are wet on submission must be dried before storage and analysis” [27]. However, it was found that excessively dry and humid conditions could decrease the amounts of drugs in the hair. In the future, the interactions between water molecules, drugs, and hair tissues should be investigated using drugs with various chemical structures to elucidate the binding mechanisms between drugs and hair tissues.

### Influence of light on drug stability in hair

The hair pigment, melanin, is decomposed by sunlight [28–30]. Artificial lights, such as fluorescent light, LED, and UV lamp, did not change hair color. Since sunlight consists of light with a wider wavelength and higher intensity than the artificial light used in this study, various lights can cause complex damage to hair tissues. Miolo et al. previously reported that amphetamines and ketamine levels decreased in hair irradiated with artificial sunlight [31]. The degradation yields depended on the compounds, light conditions, and hair properties. Additionally, changes in temperature and humidity under sunlight might also contribute to drug decrease in hair, as shown by the results of the temperature and humidity experiments (Table 1). However, this study demonstrated that all the tested drugs could be detected even in hair strands exposed to sunlight for 2 months, although the drug content in hair significantly decreased.

### Influence of soil on drug stability in hair

Casper's rule is well known in forensic medicine [32]. The decomposition rate of corpses in soil is approximately eight times slower than that in air. The results of the sunlight and soil experiments indicated a similar tendency in the decomposition rate according to this rule (Table 1). The residual rates of some drugs significantly decreased in hair strands exposed to sunlight for 2 weeks. Whereas, 2-month or longer in soil was needed to significantly decrease the amounts of drugs in the hair. Pötsch et al. reported that opiate levels in hair strands dramatically decreased after storage in soil for 6 months [33]. The decrease rate would depend on the analytes and hair properties. Additionally, various environmental conditions, such as the type of soil, season, and burial depth, contribute to the stability of drugs in hair strands buried in soil.

### Distribution of drugs in hair strands left in severe environments

It was found that 2-month of exposure to sunlight and 4-month of storage in soil significantly decreased the drug content in hair and made distribution analysis difficult (Table 2 and Fig. 3). The decrease in drug concentrations appeared to be more significant than that in the experiments described in Table 1. Because the hair strands used for the drug-distribution measurement were collected approximately 9 months after continuous ingestion of drugs, the hair strands might be damaged as compared with the reference hair strands which were collected 1 week after the final drug ingestion.

However, only slight drug peaks were detected in these severe environments. It was demonstrated that the MSA could be useful even in hair strands of corpses left in severe environments. There have been some case reports of drug detection in the hair of bodies exhumed 1 year or more after burial [34, 35]. MSA may also be applicable to the hairs of bodies exhumed to reinvestigate the bodies long after burial.

## Conclusions

In this study, the stability of drugs in hair strands left in severe environments was examined by assuming the presence of corpses in real cases. We found that the external temperature and humidity did not greatly decrease the drug content in the hair, except for DLR. Drug levels in hair significantly decreased after prolonged exposure to sunlight due to the damage of the hair surface structure and melanin. Drug levels were also decreased in the hair strands buried in soil due to decomposition of hair surface by bacteria. The rate of drug decrease was faster in hair strands exposed to sunlight than those buried in soil. However, in hair strands exposed to sunlight for 2 weeks and those buried in soil for 2 months, the distribution curves obtained using MSA were similar to those of the untreated hair strands. Moreover, in the hair strands exposed to sunlight for 2 months and those buried in soil for 4 months, drug peaks were detected in the distribution curves, reflecting the days of drug ingestion. Although the decreased rates of drug peaks depend on environmental conditions and storage time, MSA of hair strands of corpses left in severe environments can aid in the estimation of the day of death based on the compounds ingested during the lifetime of a person. Additionally, determination of intake history of specific compounds via MSA will aid in estimating the personal profiles and narrowing down the suspect list in criminal investigations.

### Supplementary Information

Below is the link to the electronic supplementary material.

Supplementary file1 (PDF 211 KB)

Supplementary file2 (DOCX 20 KB)

## Data Availability

The datasets generated during and/or analyzed during the current study are available from the corresponding author on reasonable request.
