# Supplementary material for: Effects of temperature, humidity, light, and soil on drug stability in hair: a preliminary study for estimating personal profiles using micro-segmental analysis of corpse hair
Source: Forensic Toxicol. 2023 Dec 6;42(1):60–70. doi: 10.1007/s11419-023-00675-9 (PMC10808216; doi:10.1007/s11419-023-00675-9)
Supplement: Supplementary file 1 — Supplementary file1 (PDF 211 KB) [file 11419_2023_675_MOESM1_ESM.pdf]

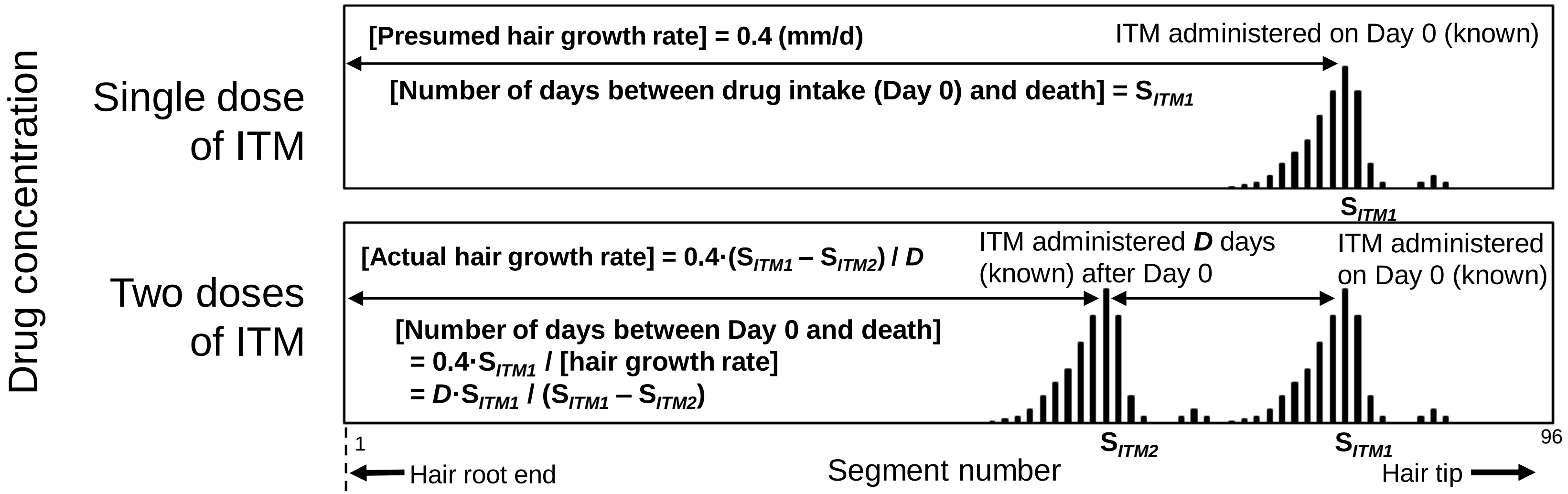

Supplementary Fig. S1 Method used to estimate the day of death using the ITM peaks

ITM, internal temporal marker; S_{ITM}, the segment number of peak corresponding to ITM ingestion

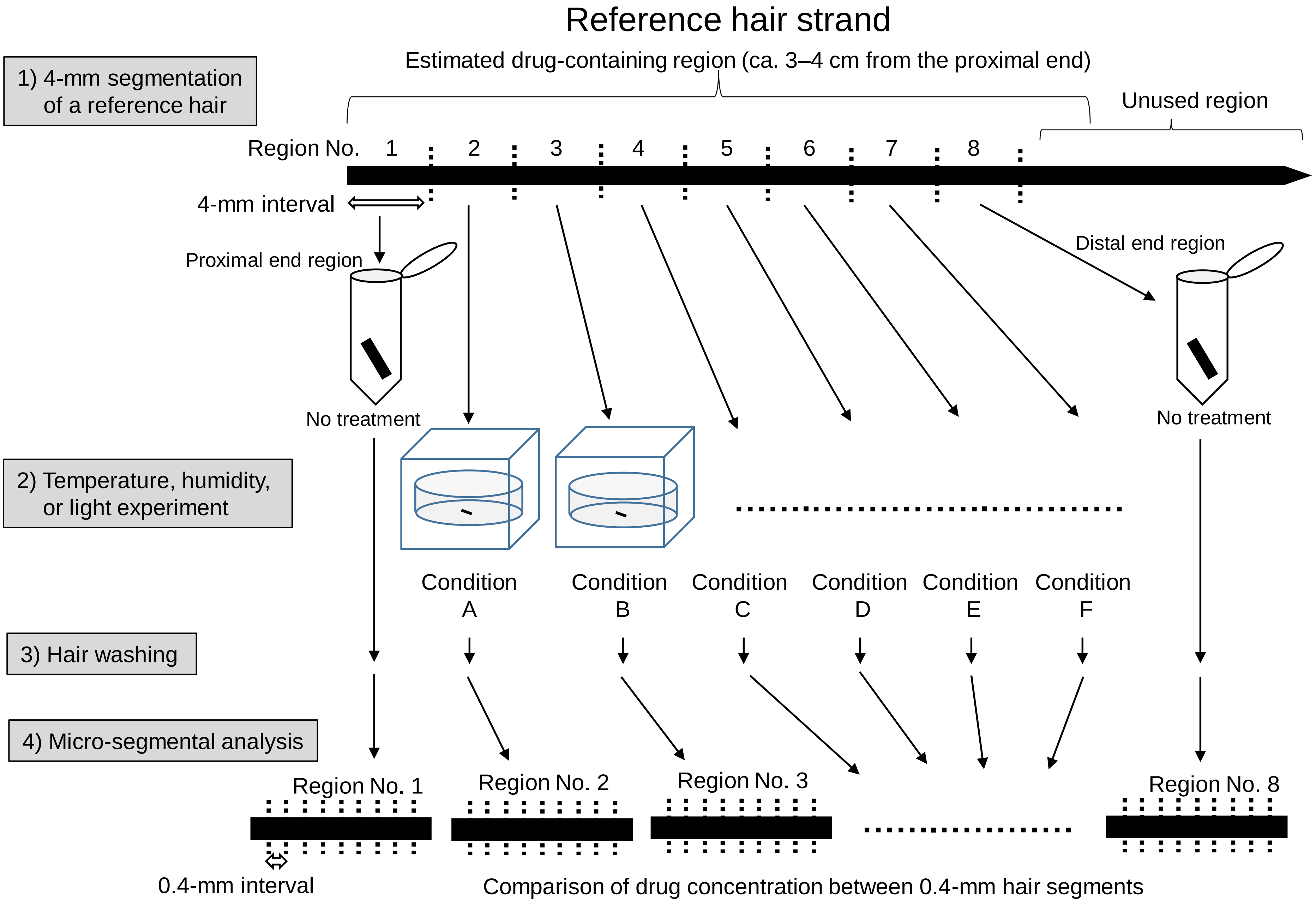

Supplementary Fig. S2 Procedures of temperature, humidity and light experiments

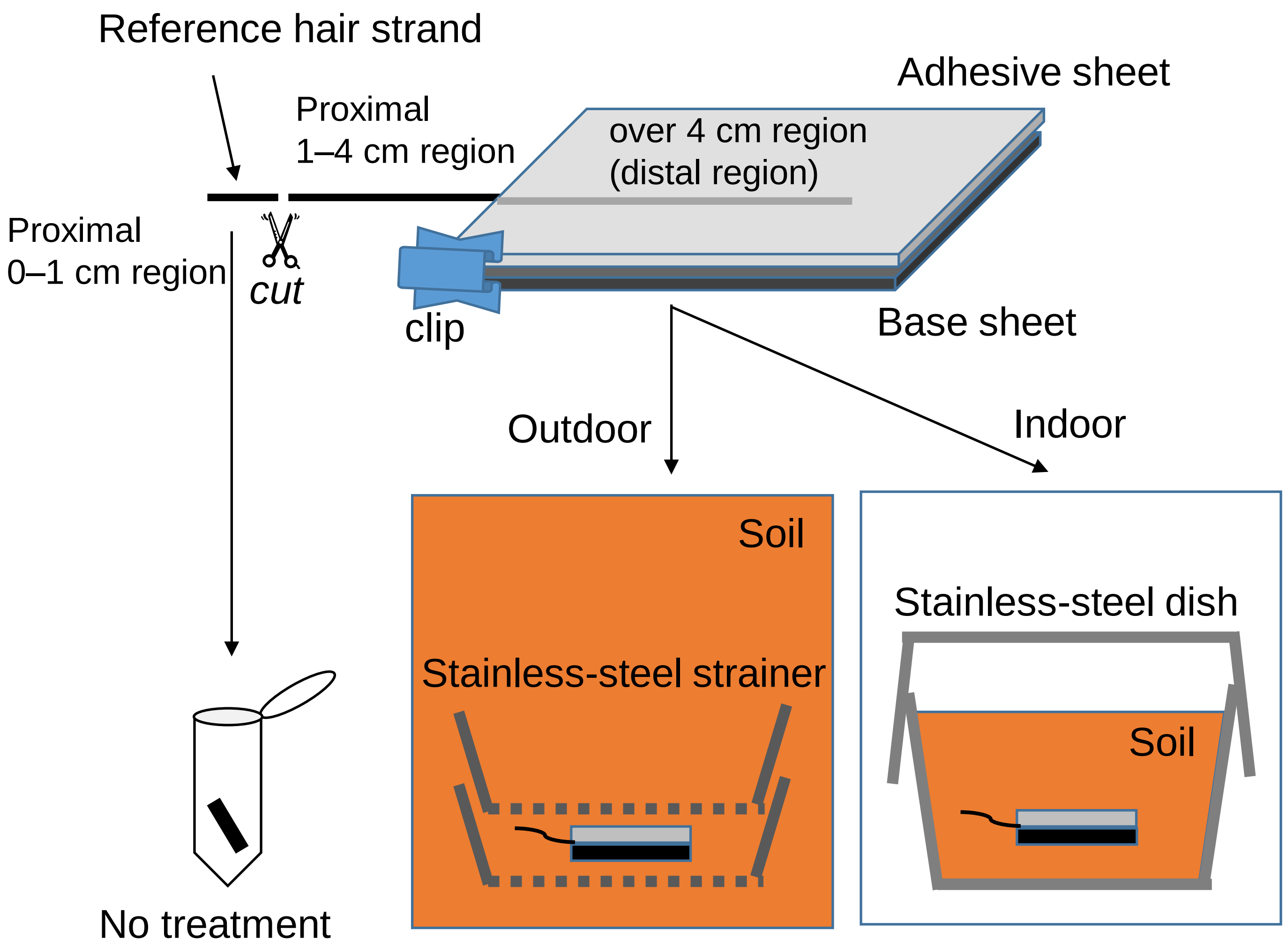

Supplementary Fig. S3 Sample preparation for soil experiments

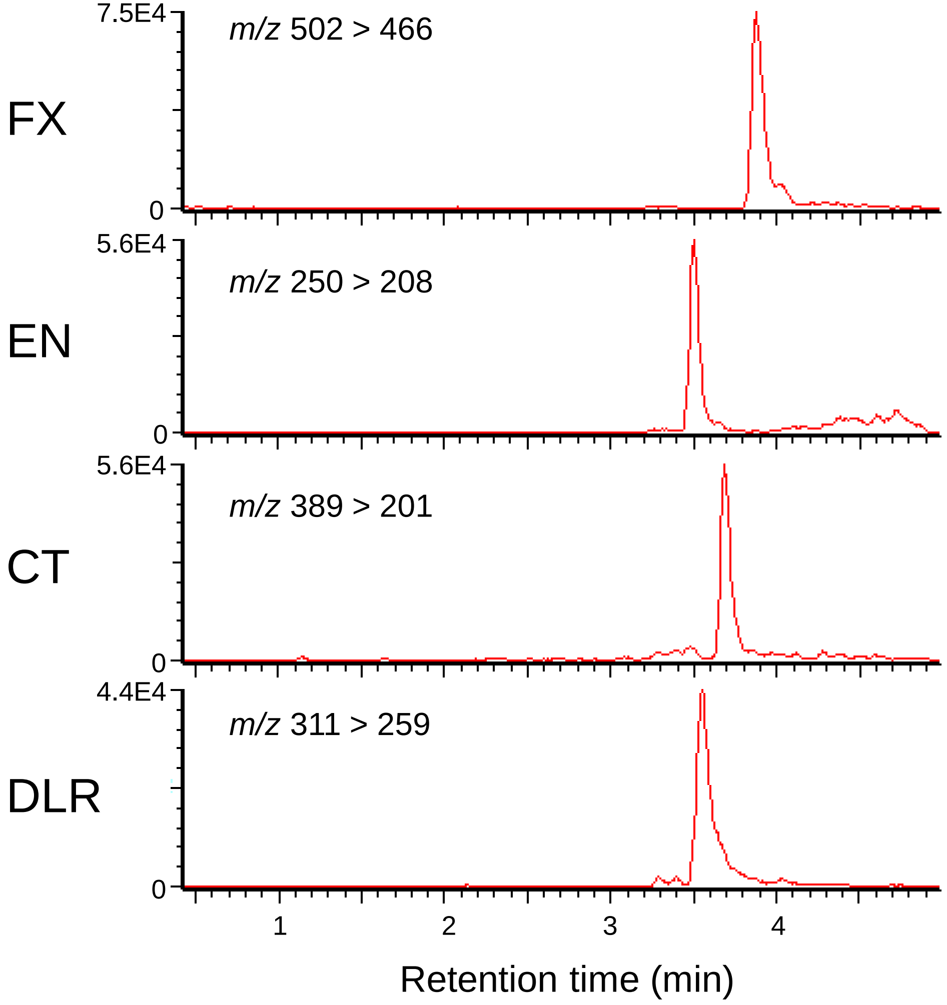

Supplementary Fig. S4 Typical SRM chromatograms of analytes extracted from a 0.4-mm hair segment

A 0.4-mm hair segment was spiked with FX, EN, CT, and DLR (100 pg/mg each).

Selected reaction monitoring (SRM), fexofenadine (FX), epinastine (EN), cetirizine (CT), desloratadine (DLR)

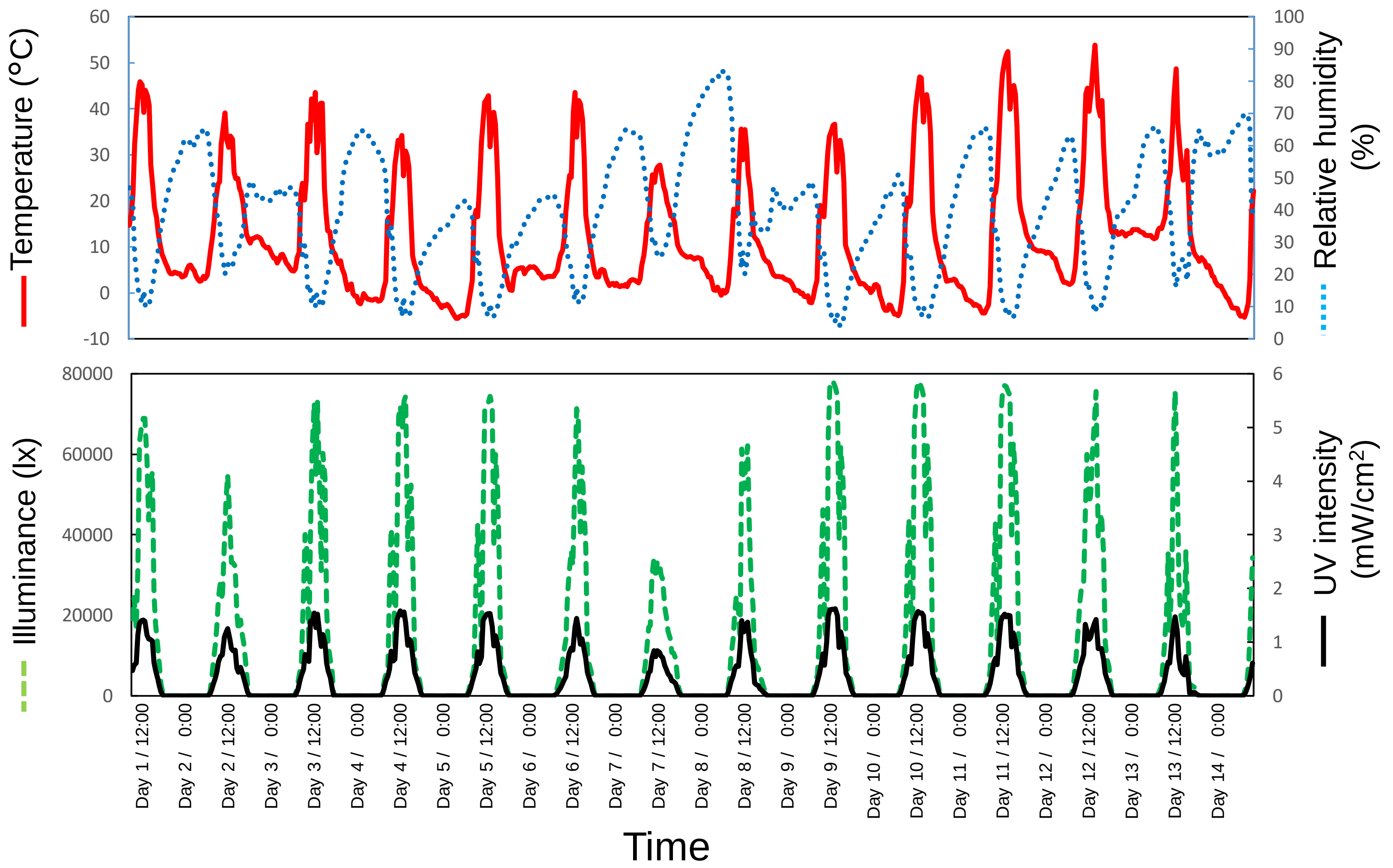

Supplementary Fig. S5 Typical time courses of temperature, humidity, illuminance, and UV intensity during the sunlight experiment

Data for the first 2 weeks of the 2-month experiment are shown.
