# Supplementary material for: Effects of temperature, humidity, light, and soil on drug stability in hair: a preliminary study for estimating personal profiles using micro-segmental analysis of corpse hair
Source: Forensic Toxicol. 2023 Dec 6;42(1):60–70. doi: 10.1007/s11419-023-00675-9 (PMC10808216; doi:10.1007/s11419-023-00675-9)
Supplement: Supplementary file 2 — Supplementary file2 (DOCX 20 KB) [file 11419_2023_675_MOESM2_ESM.docx]

Supplementary material **Table S1**. Analytical conditions for the detection of analytes in hair samples.

| LC-MS/MS | Waters ACQUITY UPLC I-Class and Xevo TQ-S |
| --- | --- |
| Column | Imtakt Cadenza CD-C18 HT (150 mm × 2.0 mm, 3 μm) |
| Column temp. | 40 °C |
| Injection volume | 50 μL |
| Flow rate | 0.2 mL/min (0–0.2 min) — 0.3 mL/min (0.2–2 min)  — 0.4 mL/min (2–4 min) — 0.5 mL/min (4–5 min) |
| Mobile phase | A: 5 mM ammonium acetate + 0.05 % formic acid  B: acetonitrile  5 % B (0–0.2 min) — 20 % B (0.2–2 min) — 50 % B (2–3 min)  — 95 % B (3–4.5 min) — 5 % B (4.5–5 min) |
| Ionization | Electrospray ionization (positive mode) |
| Capillary voltage | 3 kV |
| Desolvation temp. | 650 °C |
| Acquisition mode | SRM |
| SRM conditions | \| Analyte \| Monitoring ion  (*m/z*) \| Cone voltage  (V) \| Collision energy (eV) \| \| --- \| --- \| --- \| --- \| \| FX \| 502 > 466 \| 66 \| 26 \| \| EN \| 250 > 208 \| 10 \| 26 \| \| CT \| 389 > 201 \| 36 \| 18 \| \| DLR \| 311 > 259 \| 4 \| 20 \| \| CP-*d*_6_ \| 281 > 230 \| 2 \| 16 \| |

Liquid chromatograph-tandem mass spectrometer (LC-MS/MS), selected reaction monitoring (SRM), fexofenadine (FX), epinastine (EN), cetirizine (CT), desloratadine (DLR), chlorpheniramine (CP)

Supplementary material **Table S2**. Analytical validation using spiked 0.4-mm hair segment samples.

| Analyte | LOD  (pg mg^-1^) *^a^* | LLOQ  (pg mg^-1^) *^a^* | Spiked conc.  (pg mg^-1^) *^a^* | RE  (%)*^b^* | CV  (%)*^c^* | MF  (%)*^d^* |
| --- | --- | --- | --- | --- | --- | --- |
| FX | 1 | 5 | 5 | 13.8 | 11.7 | 111.4 |
|  |  |  | 100 | 1.6 | 9.8 | 95.6 |
| EN | 5 | 5 | 5 | -15.2 | 14.4 | 108.0 |
|  |  |  | 100 | -2.4 | 3.4 | 95.3 |
| CT | 1 | 5 | 10 | -10.3 | 2.4 | 112.3 |
|  |  |  | 100 | -1.2 | 1.0 | 110.4 |
| DLR | 5 | 10 | 10 | -9.8 | 0.8 | 71.0 |
|  |  |  | 100 | -1.0 | 1.4 | 94.4 |

Limit of detection (LOD), lower limit of quantification (LLOQ), relative error (RE), coefficient of variation (CV), matrix factor (MF)

*^a^* The weights of each 0.4-mm blank hair segment were regarded as 4 μg/segment.

*^b^* Average of 3-5 measurements

*^c^* Calculated based on measurements on 3-5 different days

*^d^* Calculated using 0.4-mm hair segments from six drug-free participants
